# Impact of Environmental Factors on Stilbene Biosynthesis

**DOI:** 10.3390/plants10010090

**Published:** 2021-01-04

**Authors:** Alessio Valletta, Lorenzo Maria Iozia, Francesca Leonelli

**Affiliations:** 1 Department of Environmental Biology, Sapienza University of Rome, Piazzale Aldo Moro 5, 00185 Rome, Italy; iozia.1602507@studenti.uniroma1.it; 2 Department of Chemistry, Sapienza University of Rome, Piazzale Aldo Moro 5, 00185 Rome, Italy; francesca.leonelli@uniroma1.it

**Keywords:** secondary metabolites, polyphenols, stilbenes, phytoalexins, biosynthetic pathway, phenylpropanoid pathway, stilbene biosynthesis, stilbene synthase, resveratrol synthase, pinosylvin synthase, environmental factors

## Abstract

Stilbenes are a small family of polyphenolic secondary metabolites that can be found in several distantly related plant species. These compounds act as phytoalexins, playing a crucial role in plant defense against phytopathogens, as well as being involved in the adaptation of plants to abiotic environmental factors. Among stilbenes, *trans*-resveratrol is certainly the most popular and extensively studied for its health properties. In recent years, an increasing number of stilbene compounds were subjected to investigations concerning their bioactivity. This review presents the most updated knowledge of the stilbene biosynthetic pathway, also focusing on the role of several environmental factors in eliciting stilbenes biosynthesis. The effects of ultraviolet radiation, visible light, ultrasonication, mechanical stress, salt stress, drought, temperature, ozone, and biotic stress are reviewed in the context of enhancing stilbene biosynthesis, both *in planta* and in plant cell and organ cultures. This knowledge may shed some light on stilbene biological roles and represents a useful tool to increase the accumulation of these valuable compounds.

## 1. Introduction

**Figure 1:**
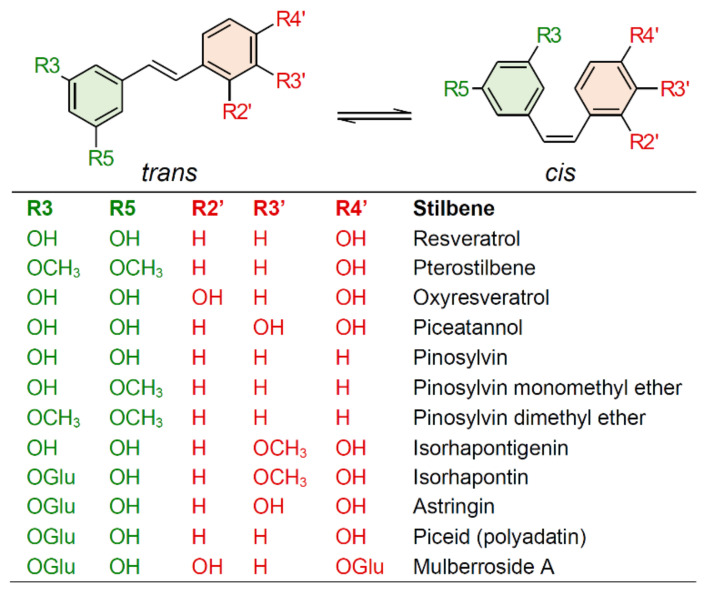
Chemical structures of common stilbene monomer derivatives. (OGlu) O-β-D-glucopyranoside.

Stilbenes are a small yet important class of non-flavonoid polyphenols, sharing a common structure characterized by a 14-carbon skeleton composed of two benzene rings linked by an ethylene bridge (Figure 1). Due to the presence of the central ethylene moiety between the aromatic rings, stilbenes exist as the two possible stereoisomers *cis* and *trans*. However, the naturally occurring stilbenes are usually in the *trans* form [1]. Plant stilbenes, together with other polyphenols such as flavonoids, isoflavonoids, curcuminoids, and xanthones, belong to the class of polyketides. Over 400 different stilbene compounds are currently known [2], mostly derived from *trans*-resveratrol (3,5,4′-trihydroxy-*trans*-stilbene) (Figure 1), although different structures can be found in specific plant families [3]. 

Stilbenes have been identified in at least 72 plant species belonging to 31 genera and 12 distantly related families, including Pinaceae (e.g., *Picea abies* (L.) Karst. and *Pinus nigra* J.F. Arnold), Gnetaceae (e.g., *Gnetum parvifolium* (Warb.) W.C. Cheng and *G. africanum* Welw.), Fabaceae (*Arachis hypogaea* L. and *Robinia pseudoacacia* L.), Vitaceae (e.g., *Vitis vinifera* L. and *V. amurensis* Rupr.), Moraceae (e.g., *Morus alba* L. and *M. macroura* Miq.), and Polygonaceae (e.g., *Polygonum cuspidatum* Sieb. et Zucc. and *P. multiflorum* Thunb.) [4,5]. Given their nutraceutical value, stilbene content and composition have mainly been investigated in food plants, and the knowledge of stilbene distribution in nature is still poor. This is partially related to the complexity of the quali-quantitative analysis of stilbenes, which is in turn related to the unavailability of standards and the detection limits of analytical methods [2]. For these reasons, most of the studies carried out to date have been focused on simple stilbenes, such as resveratrol, piceid, pterostilbene, and piceatannol (Figure 1). Current knowledge on the distribution of stilbenes in the plant kingdom will not be presented in this review, as this topic is covered by excellent recent reviews [4,5].

Stilbenes are mainly involved in constitutive and inducible protection of the plant against biotic (phytopathogenic microorganisms and herbivores) and abiotic (e.g., UV radiation and tropospheric ozone) stress [3,6]. On one side they counteract the aggression exerting a direct toxic effect on the pathogen, while on the other they act as antioxidants, protecting the cells from oxidative damage [7,8,9]. Stilbenes possess several antipathogenic properties including antibacterial, antifungal [10,11], nematocidal [12], and insecticidal [13,14]. They could also act as a deterrent towards vertebrate herbivory [15], as a possible negative effect of stilbenes has been reported on snowshoe hares (*Lepus americanus* Erxleben) [16,17] and field voles (*Microtus agrestis* L.) [18]. The role of stilbenes, among other polyphenols, in counteracting oxidative stress is just as important, as the plant response to pathogen attack involves the production of reactive oxygen species (ROS), which both act as signals for the activation of stress and defense pathways and as toxic substances capable of directly damaging the pathogen. Oxidative stress may also be induced by many abiotic conditions, such as drought, thermal stress, ultraviolet radiation, mechanical stress, heavy metals, salts, and air pollutants such as ozone [19]. Unsurprisingly, many of these factors also affect stilbene production [20].

Over the past 20 years, the bioactivities of stilbenes have been intensively investigated due to their impact on human health. Among stilbenes, resveratrol is the best known and the most studied. Basic scientific research and over 240 clinical studies have demonstrated the multiplicity of *trans*-resveratrol pharmacological effects, including antioxidant [21], anti-inflammatory [22], anticancer [23,24], estrogenic [25], neuroprotective [26], cardioprotective [27], anti-atherosclerotic [28], anti-aging [29], anti-diabetic [30], anti-osteoporosis [25], and anti-obesity properties [31]. In recent years, considerable attention has also been paid to other monomeric stilbenes, including pterostilbene [32], pinosylvin [33], and piceatannol [34], as well as to oligomeric stilbenes such as viniferins [35,36], which have been shown to possess similar and often more pronounced health-promoting properties than resveratrol. 

Due to their potential use in the nutraceutical, cosmeceutical, and pharmaceutical fields, great interest is directed at the methods for large-scale production of stilbenes. For instance, it has been estimated that the global market for *trans-*resveratrol will almost double in the next 6 years, from 58 million USD in 2020 to 99.4 million USD by 2026 [37]. Methods for obtaining stilbenes can be grouped into three categories: direct extraction from plants, chemical synthesis, and the use of biotechnologies. The chemical synthesis of stilbenes has been reported, but this method is not economically feasible, in addition to being difficult in terms of stereospecific synthesis [38,39]. Considerable efforts have been devoted to the development of biotechnological methods for stilbene production, which broadly include tissue culture techniques [40], biotransformation [41], and metabolic engineering [42]. Nevertheless, the major way of supplying stilbenes is the direct extraction from plants such as *P. cuspidatum* and *V. vinifera* [43]. 

The stilbene content and profile in stilbene-producing plants vary strongly in response to a variety of environmental factors. In recent years, a considerable body of knowledge regarding the stilbene biosynthetic pathway and the impact of environmental conditions on the production of these valuable metabolites has accumulated. This review presents the recent knowledge of the stilbene biosynthetic pathway and the impact of different environmental factors on stilbene production.

## 2. Biosynthesis of Stilbenes and Stilbenoids

**Figure 2:**
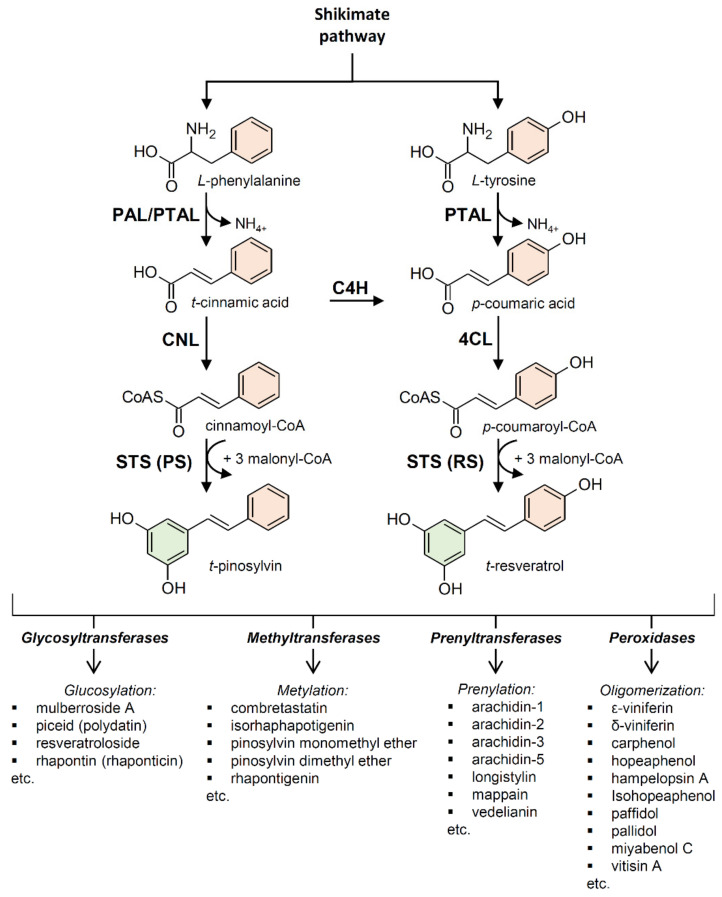
Stilbene biosynthesis in plants. (PAL) phenylalanine ammonia-lyase; (PTAL) bifunctional L-phenylalanine/L-tyrosine ammonia-lyase; (C4H) cinnamate 4-hydroxylase; (4CL) 4-coumarate:CoA ligase; (CNL) cinnamate: CoA ligase; (STS) stilbene synthase; (RS) resveratrol synthase; (PS) pinosylvin synthase.

Stilbenes and stilbenoids are biosynthesized through the phenylpropanoid pathway, which is also responsible for the biosynthesis of numerous primary and secondary metabolites including flavonoids, coumarins, hydrolyzable tannins, monolignols, lignans, and lignins [44]. Generated by the shikimate pathway, the aromatic amino acid *L*-phenylalanine is the primary starting molecule of the phenylpropanoid pathway (Figure 2). The non-oxidative deamination of *L*-phenylalanine to form *trans*-cinnamic acid, catalyzed by phenylalanine ammonia-lyase (PAL; EC 4.3.1.24), is the entry step for the carbon channeling from primary metabolism into the phenylpropanoid secondary metabolism. PAL is ubiquitous in plants [45], and it is undoubtedly the most studied enzyme involved in plant secondary metabolism [46]. Cinnamic acid can be bound to a coenzyme A (CoA) molecule by cinnamate:CoA ligase (CNL; EC 6.2.1.-) to form cinnamoyl-CoA. Alternatively, cinnamic acid can be hydroxylated by cinnamate 4-hydroxylase (C4H), a cytochrome P450 enzyme (EC 1.14.14.91), to form *p*-coumaric acid. Some plants (mainly monocots but also dicots) possess a bifunctional phenylalanine/tyrosine ammonia-lyase (PTAL, EC 4.3.1.25) that efficiently deaminates both *L*-phenylalanine (PAL activity) and *L*-tyrosine (TAL activity) [47,48,49,50]. These plants can directly produce *p*-coumaric acid using *L*-tyrosine as a substrate, bypassing the requirement for *L*-phenylalanine and C4H. A molecule of CoA is then bound to *p*-coumaric acid by 4-coumarate: CoA ligase (4CL; EC 6.2.1.12), generating *p*-coumaroyl-CoA, which provides an active intermediate in numerous branches of the general phenylpropanoid pathway [51]. 

### 2.1. Stilbene Synthase

**Figure 3:**
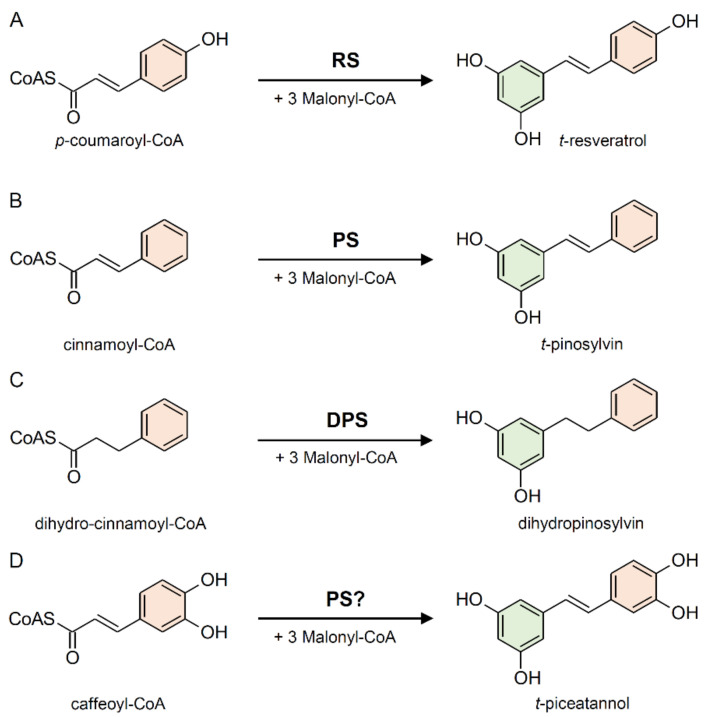
Examples of reactions catalyzed by stilbene synthase enzymes. (**A**) Conversion of *p*-coumaroyl-CoA into *t*-resveratrol by resveratrol (RS) synthase (or trihydroxystilbene synthase I). (**B**) Conversion of cinnamoyl-CoA into *t*-pinosylvin by pinosylvin synthase (PS). (**C**) Conversion of dihydro-cinnamoyl-CoA into dihydropinosylvin by dihydro-pinosylvin synthase (DPS). (**D**) Conversion of caffeoyl-CoA into *t*-piceatannol, probably catalyzed by PS.

**Figure 4:**
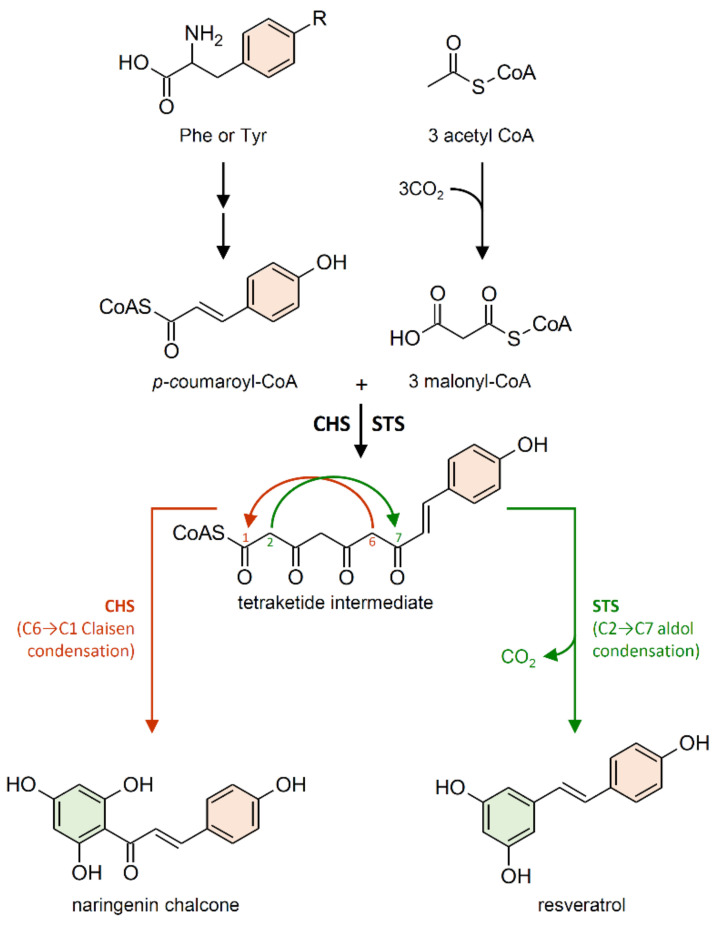
Reactions catalyzed by chalcone synthase (CHS) and stilbene synthase (STS) to produce naringenin chalcone and resveratrol, respectively. R = H phenylalanine (Phe); R = OH tyrosine (Tyr). Double arrows indicate multiple steps in the biosynthetic pathway.

The enzyme stilbene synthases (STS) catalyze the direct formation of the stilbene skeleton through a single reaction from three units of malonyl-CoA and one CoA-ester of a cinnamic acid derivative (*p*-coumaroyl-CoA to form *trans-*resveratrol or cinnamoyl-CoA to form *trans-*pinosylvin) [52] (Figure 2 and Figure 3). Malonyl-CoA is generated through a carboxylation reaction between acetyl-CoA and a bicarbonate ion (HCO_3_^−^) catalyzed by acetyl-CoA carboxylase (EC 6.4.1.2) in the presence of ATP (Figure 4).

Based on the preferred starting substrate, STS enzymes are classified into either a *p*-coumaroyl-CoA-specific type, such as trihydroxystilbene synthase I (also known as resveratrol synthase, EC 2.3.1.95), or a cinnamoyl-CoA-specific type, such as pinosylvin synthase (EC 2.3.1.146) (Figure 2 and Figure 3). The former type has been mainly found in angiosperms like peanut [53], grapevine [54], and Tatar rhubarb (*Rheum tataricum* L.f) [55], while the latter type is typical in conifers and has been identified in several *Pinus* species like Scots pine (*P. sylvestris* L.) [56], Japanese red pine (*P. densiflora* Siebold & Zucc.) [57], and Eastern white pine (*P. strobus* L.) [58]. 

*Pinus* species can biosynthesize two types of stilbenes, i.e., pinosylvin and dihydropinosylvin, which are biosynthetically derived from cinnamoyl-CoA and dihydrocinnamoyl-CoA, respectively (Figure 3B,C). STS from *P. strobus* shows a clear preference for cinnamoyl-CoA and was therefore characterized as pinosylvin synthase [58]. Otherwise, STS from *P. sylvestris* shows an unusual preference for dihydro-cinnamoyl-CoA, identifying it as a dihydro-pinosylvin synthase [56]. STS does not exhibit absolute substrate specificity. While showing a preference for a given substrate, the same STS enzyme can accept different cinnamic acid derivatives as starting substrates catalyzing the biosynthesis of different stilbenes. For example, the enzyme responsible for the biosynthesis of piceatannol (3,5,3′,4′-tetrahydroxystilbene) has not been identified yet, however, pinosylvin synthase from *P. strobus* proved to be active with caffeoyl-CoA in vitro (Figure 3D), suggesting that it could be responsible for piceatannol biosynthesis *in planta* [58]. 

STS enzymes belong to the type III polyketide synthase superfamily (PKSs), which also includes chalcone synthase (CHS; EC 2.3.1.74) [59]. STS and CHS share a high degree of similarity both in their amino acid sequence identity (which reaches 75–90% depending on the species) and in their crystallographic structures [51,60]. *CHS* genes are present in the genome of all plants analyzed so far, while *STS* have been identified in a limited number of plant species, often phylogenetically unrelated. Converging lines of evidence indicate that CHS is the archetypal enzyme from which STS evolved multiple times independently in stilbene-producing plants, through gene duplication followed by functional divergence [60,61,62]. CHS and STS are the most investigated enzymes among PKSs and, due to their high sequence similarity, they are often referred to as the CHS/STS family [63,64].

Although it employs the same substrates as STS, CHS is responsible for the first committed step in the biosynthesis of flavonoid-type compounds. Both enzymes generate the same linear tetraketide intermediate. However, CHS catalyzes a C6→C1 Claisen condensation of the intermediate to produce naringenin chalcone, while STS catalyzes an alternative C2→C7 aldol condensation of the intermediate to form a stilbene backbone (Figure 4) [59,65,66]. 

STS was first extracted and purified from suspension cultures of peanut cells elicited with UV radiation [53]. Cloning of two peanut *STS* genes revealed a high sequence identity with *CHS* throughout the coding region and the presence of an intron at the same position as a conserved intron in *CHS* [67]. *STS* genes and cDNAs were subsequently cloned and characterized from grapevine cell suspension cultures [68] and Scots pine plantlets [56], both induced by fungal elicitors. At present, *STS* genes have been cloned from several plant species including mulberry (*Morus notabilis* C.K. Schneid and *M. atropurpurea* Roxb.) [42,66], Scots pine [69], white spruce (*Picea glauca* (Moench) Voss) [70], Norway spruce (*Picea abies* (L.) H. Karst.) [71], Japanese red pine [57], and sorghum (*Sorghum bicolor* (L.) Moench) [72]. To the best of our knowledge, sorghum is the only monocot plant in which an *STS* gene (*SbSTS1*) has been identified. 

In most stilbene-producing plants, *STS* exists as a small family consisting of 1–10 closely related paralogs. For example, the *STS* multigene family is represented by two members in white spruce [70] and Norway spruce [71], three members in Japanese red pine [57], almost five members in Scots pine [69], six members in peanut [73], and ten members in mulberry [66]. Remarkable exceptions to this role are sorghum, in whose genome only one *STS* gene has been identified [74,75], and grapevine, which possesses an uncommonly large number of *STS* genes. Both grapevine and sorghum genomes have been entirely sequenced [74,76]. Early Southern-blot analysis suggested that the grapevine *STS* gene family consisted of 15–20 members [77]. Genome-wide analysis carried out on the *V. vinifera* PN40024 genome led to the identification of 48 putative *STS* genes, designated *VvSTS1* to *VvSTS48*, with at least 33 potentially coding for functional STS proteins [60,62]. 

To date, there is no evidence regarding the different substrate specificity and enzymatic activity of different *VvSTS*s. Functional characterization of nine *VvSTS*s confirmed that they encode for functional STS enzymes [62]. Since these nine genes were specifically chosen to represent the diversity of the *VvSTS* gene family, it is most likely that all grapevine *VvSTS*s encode enzymes with similar activity and specificity. Despite the high similarity between *STS* genes which makes it difficult to accurately distinguish the individual transcripts, gene expression studies revealed different transcriptional responses of distinct *VvSTS*s during development and in response to environmental stresses [60,78,79]. The expression of some *VvSTS*s was also found to be tissue-specific [60,79]. It is therefore likely that the large quantity of members in the grapevine *STS* gene family has evolved to allow for fine spatial and temporal regulation of stilbene biosynthesis under both normal and stress conditions.

### 2.2. Glucosylation/Deglucosylation

Glycosylation is one of the most common modifications of plant secondary metabolites [80,81] that can modify their physicochemical and biological properties. Water-solubility, physicochemical stability, biological half-life, compartmentalization, and biological activity of stilbenes and other phenylpropanoids can be dramatically altered by glycosylation [82,83,84]. 

**Figure 5:**
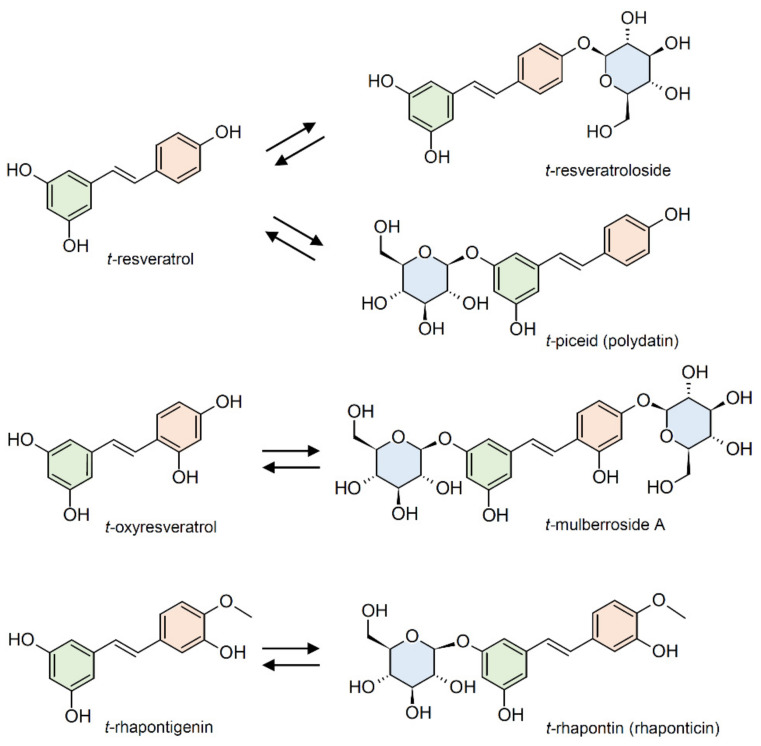
Common examples of stilbene glucosylation.

In stilbene-producing plants, a significant fraction of stilbenes is accumulated in a glucosylated form [85]. For instance, *Fallopia japonica* Houtt. (formerly *Polygonum cuspidatum*) produces both resveratrol and resveratrol 3-*O*-*β*-glucoside (commonly known as piceid or polydatin) (Figure 5), and the glucosylated form can reach concentrations of up to six times higher than the free aglycone [86]. *Morus alba* L. and *Rheum undulatum* L. accumulate the glucosylated stilbenes mulberroside A (a diglucoside of oxyresveratrol) and rhapontin (a monoglucoside of rhapontigenin, also known as rhaponticin) [87] (Figure 5). Significant amounts of *cis*- and *trans*-piceid are accumulated in grapevine, both constitutively [40,88,89] and in response to pathogen attack [90,91] and to environmental stresses such as UV light [88,92,93,94], salinity [95], and drought [96,97].

Numerous glycosyltransferases that produce glucose esters of hydroxybenzoic and hydroxycinnamic acids accept a broad spectrum of structurally similar substrates [98]. A bi-functional glycosyltransferase from Concord grape (*Vitis labrusca* L.) (VLRSgt) that produces stilbene glucosides and glucose esters of hydroxycinnamic acids in vitro has been characterized in 2007 by Hall and De Luca [99]. The mesocarp-specific expression of VLRSgt reflected the increased accumulation of resveratrol glucosides during berry maturation, coherent with a role for this enzyme in stilbene glucosylation in the mesocarp.

It is well known that glucosylation increases resveratrol water solubility [82] and helps to protect stilbenes and other polyphenols from enzymic oxidation [100,101], which could extend their half-life in plant cells, preserve their biological properties, and assist stilbene transportation and accumulation.

Considerable levels of *trans*-piceid can be found in grape derivatives such as wines and juices [102,103]. Due to the β-glucosidase activity of yeasts, a decrease in *trans-*piceid concentration accompanied by an increase in *trans-*resveratrol concentration is often observed during grape fermentation [104,105]. Since *trans-*resveratrol has better health properties than piceid, a great deal of interest has been paid to strategies aimed to increase *trans-*resveratrol concentration during winemaking. One of these consists in the selection of yeast strains with high β-glucosidase activity, capable of efficiently converting *trans-*piceid into the free aglycone during the alcoholic fermentation [106,107]. Bacterial or fungal β-glucosidases can also be used to obtain *trans-*resveratrol from plant extracts rich in piceid, such as those obtained from *P. cuspidatum* [108,109]. Other glycosylated stilbenes, such as mulberroside A and rhaponticin from *M. alba* and *R. undulatum*, can be enzymatically converted to their aglycones oxyresveratrol and rhapontigenin with an increase in their bioactivity [87,110,111,112].

### 2.3. Methylation

**Figure 6:**
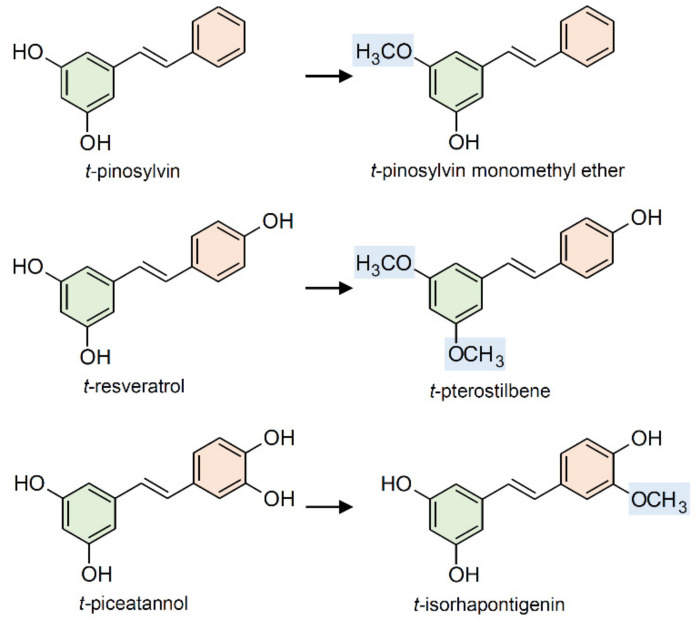
Common methylated stilbenes are biosynthetically derived from pinosylvin, resveratrol, and piceatannol.

Methylation of stilbene hydroxyl side groups leads to the formation of methoxystilbenes (Figure 6). Among the most known methoxystilbenes, there is pinosylvin monomethyl ether (3-hydroxy-5-methoxystilbene) found in several *Pinus* spp. [113,114] and *Alnus* spp. [115], and pterostilbene (3,5-dimethoxy-4′-hydroxystilbene), biosynthesized by red sandalwood (*Pterocarpus santalinus* Lf) [116], Indian Kino (*Pterocarpus marsupium* Roxb.) [117], *Vaccinium* spp. berries [118], and, at low levels, in grapevine leaves and berries [92,93,94,95,96,97,98,99,100,101,102,103,104,105,106,107,108,109,110,111,112,113,114,115,116,117,118,119]. Methylation of stilbenes, as well as of several other thousands of plant secondary metabolites, is catalyzed by *S*-adenosyl-L-methionine (SAM)-dependent *O*-methyltransferases (OMTs; EC 2.1.1). 

Methylation of hydroxyl groups alters the solubility and reactivity of stilbenes and can therefore affect their biological activity. For instance, pterostilbene has been shown to be 5–10 times more active than non-methylated resveratrol in inhibiting the germination of downy mildew (*Plasmopara viticola*) sporangia and grey mold (*Botrytis cinerea*) conidia [120]. Pinosylvin monomethyl ether has been reported to have significantly lower antifungal and antibacterial activity than pinosylvin [121], although it has shown greater activity against some brown-rot fungi [122]. 

Investigations on the relationship between chemical structure and biological activity revealed increased cytotoxicity and anticancer activity associated with resveratrol methylation [123,124]. The substitution of hydroxy with methoxy groups enhances the lipophilicity of pterostilbene over resveratrol, which results in high bioavailability. This difference in pharmacokinetics might explain the higher bioactivity of pterostilbene over its parental compound resveratrol. Methylated resveratrol derivatives have consequently become attractive target compounds for both bioproduction and metabolic engineering [125,126].

Several plant OMTs have been characterized in the last decades. However, the majority of them have been found to be involved in the methylation of aromatic hydroxyl groups of different compounds such as benzylisoquinoline alkaloids [127], phenylpropanoids [128], and flavonoids [129], while only a few stilbene-specific OMTs have been reported to date.

*V. vinifera* resveratrol OMT (VvROMT) was shown to specifically catalyze the methylation of resveratrol and pinosylvin (3,5-dihydroxystilbene) at the C-3 or C-5 positions [130]. VvROMT was shown to convert resveratrol to pterostilbene both in vitro and *in planta*. The transient co-expression of *VvROMT* and *VvSTS* in tobacco resulted in the accumulation of pterostilbene. *VvROMT* gene expression in grapevine leaves was induced by different stresses, including *P.* *viticola* infection and UV radiation, accordingly with the role of pterostilbene in chemical plant defense [130]. *S. bicolor* resveratrol OMT (SbOMT1) has been shown to catalyze the 4′-*O*-methylation of resveratrol both in vitro and *in planta* [125,131]. In 2019, Koeduka and colleagues [132] isolated and characterized a putative aromatic *O*-methyltransferase gene (*AcOMT1*) in *Acorus calamus* (Araceae) using RNA-seq analysis. Recombinant AcOMT1 expressed in *Escherichia coli* showed high 4′-*O*-methylation activity toward resveratrol and its derivative, isorhapontigenin (3,4′,5-trihydroxy-3′-methoxystilbene). 

In Scots pine, pinosylvin can be methylated by a pinosylvin O-methyltransferase (PsPMT1) to pinosylvin monomethyl ether, following ozone or fungal elicitation treatment [133]. However, it should be noted that PsPMT1 showed a relatively broad substrate specificity, methylating several compounds such as stilbene aglycones, flavonoids, and hydroxycinnamic acids, many of these even more efficiently than pinosylvin. In 2017, Paasela and co-workers [134] subsequently isolated and characterized an *O*-methyltransferase from *P. sylvestris* (PpPMT2), which is held responsible for the methylation of pinosylvin. Unlike the multifunctional PsPMT1, PsPMT2 preferentially methylated pinosylvin into its monomethyl ether, showing a high degree of specificity for stilbenes. The authors observed that PsPMT2 is co-expressed with STS in response to wounding of xylem and UV-C treatment of needles, suggesting that these two enzymes are under common regulation.

### 2.4. Prenylation 

Prenylated stilbenoids have been isolated from a restricted number of stilbene-producing plants including *Macaranga* spp. (Euphorbiaceae) [135,136], *Glycyrrhiza* spp. [137,138,139,140], peanut [141,142,143,144,145,146], and mulberry [147]. 

**Figure 7:**
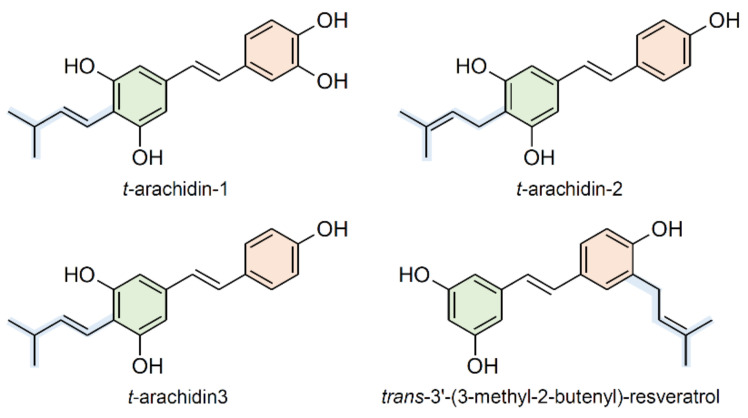
Major prenylated stilbenoids contained in peanuts.

Over 45 prenylated stilbenoids have been identified in *A. hypogaea* [148]. The major prenylated stilbenoids accumulated in peanuts are *trans-*arachidin-1, *trans-*arachidin-2, *trans-*arachidin-3, and *trans*-3′-(3-methyl-2-butenyl)-resveratrol (Figure 7) [148]. In accordance with their role as phytoalexins, peanut prenylated stilbenoids have been shown to accumulate upon challenge with microorganisms [142,143,144,145,149] and to possess remarkable antifungal activity [150,151,152]. Several recent studies have shown interesting therapeutic potential for peanut prenylated stilbenoids [135,136,153,154]. Prenylated compounds generally exhibit greater bioavailability than their non-prenylated counterparts, due to the increase in lipophilicity linked to the prenyl groups. Despite their biological and medical relevance, the biosynthetic pathways of prenylated stilbenoids have yet to be elucidated and the genes encoding stilbenoid-specific prenyltransferases have only recently been identified in plants. 

In 2018, membrane-bound stilbene-specific prenyltransferases have been described in peanut and mulberry [148,155]. Combining targeted transcriptomic and metabolomic analyses, Yang et al. [148] discovered five candidate prenyltransferase genes in elicitor-treated *A. hypogaea* hairy root cultures. Two of these, *AhR4DT-1* and *AhR3DT-1*, were functionally characterized in a transient expression system consisting of *Agrobacterium*-infiltrated leaves of *Nicotiana benthamiana* Domin. The authors demonstrated that AhR4DT-1 catalyzes the prenylation of resveratrol at its C-4 position leading to arachidin-2 formation, while AhR3DT-1 is responsible for resveratrol prenylation at C-3′ leading to the 3-methyl-2-butenyl-3-resveratrol formation. In 2018, Zhong and colleagues [155] identified and functionally characterized a stilbenoid-specific prenyltransferase from *M. alba* (MaOGT) that recognizes oxyresveratrol and geranyl diphosphate (GPP) as natural substrates and catalyzes oxyresveratrol prenylation. Both peanut and mulberry prenyltransferases have proved to be highly specific for stilbene substrates and fluorescent microscopy analysis has shown that they are localized in the chloroplast, similarly to other membrane-bound plant prenyltransferases [156].

### 2.5. Oligomerization

Stilbenes are often accumulated in plants as oligomers (oligostilbenes) resulting from the oxidative coupling of stilbene monomers [157,158]. Oligostilbenes have been isolated from species belonging to different plant families including Vitaceae [159], Fabaceae [160], Cyperaceae [161], Dipterocarpaceae [162], Gnetaceae [163], Paeoniaceae [164], Iridaceae [165], and Moraceae [166]. The interest addressed to oligostilbenes in recent decades is linked to their important biological role in the plant as phytoalexins [167], to their chemical diversity with more than 200 different molecules known to date [158], and to the wide spectrum of biological activities such as β-secretase inhibitory, anti-influenza virus, and anti-herpes simplex virus activities [168].

**Figure 8:**
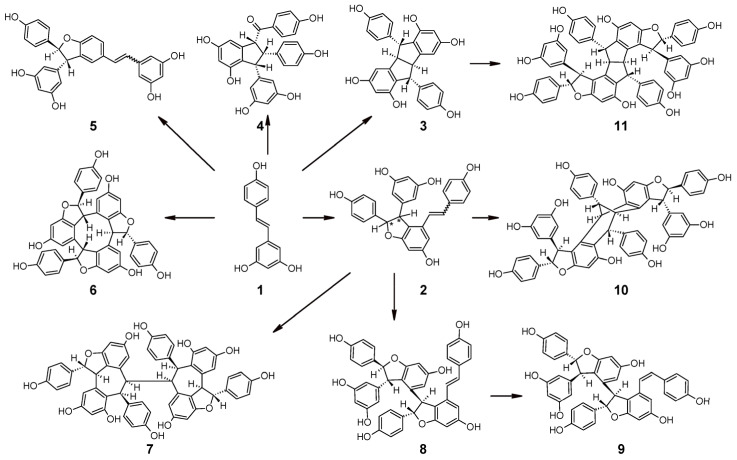
Of resveratrol trimers and tetramers in grapevine: (**1**) *trans*-resveratrol; (**2**) (E and Z) ε-viniferin/ω-viniferin; (**3**) pallidol; (**4**) caraphenol B; (**5**) δ-viniferin (E and Z); (**6**) α-viniferin; (**7**) isohopeaphenol; (**8**) E-miyabenol C; (**9**) Z-miyabenol C; (**10**) vaticanol C isomer; and (**11**) ampelopsin H [181,182].

The largest group of oligostilbenes is represented by resveratrol oligomers, arising from the polymerization of two to eight resveratrol units [169,170] (Figure 8). Among these, the most investigated are viniferins, which accumulate in *V. vinifera* upon abiotic stress (e.g., UV irradiation) or fungal infection (e.g., *Botrytis cinerea* and *Plasmopara viticola*) [8,120,171]. 

Resveratrol oligomerization has been achieved in vitro by enzymatic oxidation utilizing horseradish peroxidase [172,173] or laccase-like stilbene oxidase from *B. cinerea* [174,175]. It has been proposed that peroxidases (POD or POX EC 1.11.1.7) located in the plant cell wall and vacuole are responsible for oxidative polymerization of resveratrol to form its natural oligomers [157,176]. POD enzymes use hydrogen peroxide to catalyze oxidative reactions and have already been exploited for the in vitro synthesis of oligostilbenes [173,177,178]. However, it should be noted that in vitro experiments with these enzymes did not lead to the formation of oligomers with the natural configuration found in plants, and to date, there is no direct evidence of the involvement of specific peroxidases in the formation of oligomeric stilbenes *in planta* [5].

In recent years, considerable efforts have also been made to develop non-enzymatic methods to produce oligostilbenes [179,180].

## 3. Impact of Environmental Factors on the Biosynthesis of Stilbenes

It is known that the biosynthesis of stilbenes in plants can be triggered by a variety of biotic and abiotic environmental factors. Some of these, like fungal infection and UV-C radiation, have been under study for many years, while others like bacterial infection and ozone stress have only recently caught the attention of the research community. In Table 1 and in the following paragraphs some of the most important results coming from studies on the influence of different environmental factors on the biosynthesis of stilbenes are reported and described.

### 3.1. UV Radiation

Multiple lines of evidence indicate that stilbenes, as well as other polyphenols, play an important role in protecting plants from the damaging effects of ultraviolet (UV) radiation [243,244,245]. Induction of polyphenolic phytoalexin biosynthesis in response to UV exposure has been observed in numerous plants [246,247]. Non-polyphenolic phytoalexins can also be elicited by UV, e.g., labdane-related diterpenoids in rice [248] and terpenoid indole alkaloids in the Madagascar periwinkle (*Catharanthus roseus* (L.) G.Don) [249].

The solar UV spectrum is conventionally subdivided into three wavelength ranges: UV-A (315–400 nm), UV-B (280–315 nm), and UV-C (100–280 nm) [250]. UV-C is extremely harmful to organisms, but it is naturally filtered by the stratospheric ozone layer and it is consequently not relevant under natural conditions of solar irradiation. Ozone absorption coefficient drops rapidly at wavelengths longer than 280 nm, reaching zero around 330 nm, therefore UV-B and UV-A can reach Earth’s surface and interact with plants [251]. However, UV harmfulness declines in a similar way, as UV-A cannot be absorbed by DNA and it is thus far less damaging for plants [252]. As a result, for a long time, the effects of UV-A on plant physiology had been underestimated and most studies have focused primarily on UV-C and subordinately on UV-B.

UV-C significantly increases stilbene production in different stilbene-producing plants including grapevine [92,184,194,253], peanut [216,243,254], *Gnetum parvifolium* (Warb.) W.C. Cheng [204,205], *Picea jezoensis* (Siebold & Zucc.) Carr. [255], and *Polygonum cuspidatum* Siebold and Zucc. [256] (Table 1). Recently, Vannozzi and colleagues [60] performed a genome-wide analysis of the *VvSTS* multigene family expression pattern on *V. vinifera* cv. Pinot Noir (‘PN40024’ genotype) leaf disks subjected to different stresses (i.e., UV-C exposure, wounding, and downy mildew infection) through a whole transcriptomic (RNA-seq) and real-time qRT-PCR approach. They observed that all stress treatments led to a significant up-regulation of at least several members of the *VvSTS* multigene family. However, UV-C exposure resulted in the highest induction of the majority of *VvSTS* members. The induction of *VvSTSs* was accompanied by reduced expression of *VvCHS* genes to lower levels compared to untreated leaf discs, suggesting that the competitive relationship between *VvSTS* and *VvCHS* may play a role in the accumulation of stilbenes in response to UV-C [60,198].

Molecular mechanisms behind the induction of stilbene biosynthesis in response to UV radiation are still not completely understood. The induction is accompanied by transcriptional activation, protein accumulation, and activation of STS and other enzymes involved in stilbene biosynthesis [5]. In 2013, Höll and co-workers [257] reported that two R2R3-MYB–type transcription factors, namely *MYB14* and *MYB15*, control the transcriptional expression of *VvSTS* genes under UV-C irradiation in *V. vinifera* cv. Shiraz leaf discs. However, these transcription factors appear not to be specifically involved in response to UV-C, but also to other stresses including fungal infections and wounds. 

In a recent study, the member of the stilbene synthase family *VpSTS29* derived from Chinese wild *Vitis pseudoreticulata* W.T. Wang was overexpressed in *V. vinifera* cv. Thompson Seedless and the localization of the VpSTS29-GFP protein was investigated [247]. The accumulation of stilbenes elicited by UV-C irradiation was accompanied by the translocation of the VpSTS29-GFP protein from the cytoplasm to the chloroplast. Interestingly, transgenic plants overexpressing VpSTS29-GFP exhibited much lower H_2_O_2_ content than untransformed plants and an altered expression of genes related to redox processes, stilbene biosynthesis, and light stimulus [247].

Regarding UV-B, several studies showed that these wavelengths can stimulate the biosynthesis of polyphenols, including stilbenes [184,199,200,258]. UV-B triggers a radiation-specific signaling pathway in grape skin, which activates the biosynthesis and accumulation of secondary metabolites [259]. A UV-B treatment was found to elicit a significant increase in stilbene production, although less marked than that achieved with UV-C [260]. In 2008, Berli and co-workers [258] analyzed the stilbene content in berry skins collected from plants of *V. vinifera* cv. Malbec cultivated under sunlight with full UV-B (+UV-B) or filtered UV-B (−UV-B) in three different locations at 500, 1000, and 1500 m above sea level (asl) and observed that different solar UV-B levels affect the accumulation of *trans-*resveratrol. The highest resveratrol content was detected in the berry skins from the +UV-B treatment at 1500 m asl, where the difference between +UV-B and −UV-B was statistically significant. Studies on ozone-treated Scots pine also showed a slight increase in stilbene synthase mRNA, as well as in pinosylvin and pinosylvin methyl ether contents under exposure to UV-B light [261,262].

Post-harvest treatment with UV-B and UV-C has been widely used in fruit and vegetable storage [199,263], where it delays fruit ripening and senescence [264], and activates the defenses against pathogens [265]. Post-harvest UV treatment has been exploited in grapes to increase the content in phenolic compounds including stilbenes in berries and wines [199] (Table 1). 

As previously mentioned, the impact of UV-A on stilbene biosynthesis has been underestimated, however recent studies suggest that this radiation deserves more attention. An increase in stilbene content related to UV-A irradiation has been observed in *Eucalyptus nitens* (H. Deane and Maiden) Maiden by Close and colleagues [266]. The authors postulated that, since stilbenes have absorbance properties consistent with a function as UV-A screens, they could be part of an active UV-A response. This conclusion is consistent with the observation that adult retinal pigment epithelial cells treated with resveratrol show higher viability when exposed to UV-A [267]. A role of UV-A in stilbene biosynthesis was observed in leaves of grey alder (*Alnus incana* (L.) Moench) and white birch (*Betula pubescens* Ehrh.) trees under field conditions [268]. It was recently observed that *trans*-resveratrol and *trans*-pterostilbene biosynthesis in leaves of O’Neal high bush blueberries (*Vaccinium corymbosum* L.) can be enhanced by irradiation with both UV-A and UV-C [269]. The comparison of the two UV-treatments showed that UV-A is more effective in promoting *trans-*resveratrol production, while UV-C is better in enhancing *trans*-pterostilbene production. 

### 3.2. Light 

Light is vital for plants, representing the main energy source for these phototrophic organisms. Light plays a pivotal role in plant growth and development, but it also affects secondary metabolism [20,270]. Extensive literature shows the relationship between light and biosynthesis of polyphenol compounds such as anthocyanins and flavonols [271,272]. However, only a limited number of studies are available on the light-dependent regulation of the stilbene biosynthetic pathway.

Advancements in technology have recently brought light-emitting diodes (LEDs) to the scene of botanical research, allowing for the use of specific wavelengths at high irradiance levels, with realistic results on the study of plant physiological responses to them [273]. Although there is still scarce information about stilbene biosynthesis in response to LED lighting, several studies observed an increase in stilbene biosynthesis, as well as in the expression of stilbene biosynthetic genes, in grapevine exposed to specific wavelengths.

The influence of red LED light (625 nm) and methyl jasmonate (MeJa) on the production of phenylpropanoids in *V. vinifera* cv. Barbera cell suspension cultures were investigated by Tassoni and co-workers [211]. The combined treatment with red light and MeJa increased the biosynthesis of both anthocyanins and stilbenes, while also promoting the release of catechins into the culture medium. The treatment with red light alone produced a 50% increase in stilbene content in grapevine cells, accompanied by an average decrease in anthocyanin and catechin content of 10% and 18%, respectively. These results suggest a diversion of the phenylpropanoid pathway towards the production of stilbenes under red light [211].

A series of experiments on berries of *V. labruscana* Bailey cvs. Campbell Early and Kyoho irradiated with fluorescent white light or purple (380 nm), blue (440 nm), and red (660 nm) LED lights showed that red and blue light induces the upregulation of several *STS* genes and the accumulation of *trans-* and *cis*-resveratrol, *trans*- and *cis*-piceid, and piceatannol both in grape berry skin [212], and in detached leaves [213]. 

In 2015, Taurino and colleagues [214] observed an inhibitory effect of light on *trans*-resveratrol production and an enhancement of *trans*-piceid biosynthesis in cell suspension cultures of *V. vinifera* cv. Negramaro. Moreover, in 2018, Andi and collaborators [215] reported that high-level white light irradiation (10,000 lux) inhibits the biosynthesis of *trans*-piceid and *trans*-resveratrol in cell suspension cultures of *V. vinifera* cv. Shahai.

Interesting differences in the impact of light on constitutive and MeJa-induced stilbene biosynthesis emerged from the comparison of cell lines of *V. vinifera* cv. Malvasia and *V. rupestris* Du Lot [40]. In both species, the constitutive stilbene content was higher under light conditions, although *V. vinifera* mainly accumulated piceid, while *V. rupestris* accumulated *trans*-resveratrol, *trans*-δ-viniferin, and *trans*-ε-viniferin. Furthermore, *V. vinifera* cells responded to MeJa elicitation with a significant increase in stilbene production under both light and dark conditions, while *V. rupestris* cells were responsive to elicitation exclusively under dark conditions [40].

Research has only recently begun to investigate the relationship between light and stilbene biosynthesis in species other than grapevine. A study on *A. hypogaea* sprouts treated with UV-C and white LED light showed a significant response to white light in stilbene accumulation by the upregulation of genes and enzymes involved in their biosynthetic pathway, although less prominent than that observed in response to UV-C [216].

### 3.3. Temperature

Temperature is an environmental factor of primary importance for plants. It is among the most crucial climatic drivers of biodiversity [274,275] and significantly affects gene expression, protein synthesis, enzymatic activity, and overall primary and secondary metabolism [276,277,278]. Numerous studies have investigated the influence of temperature on the biosynthetic pathway of polyphenols, but only a small fraction of these have focused on stilbenes [204,205,206,207] (Table 1).

A recent study on *Gnetum parvifolium* revealed that exposure to high temperature (40 °C) enhances resveratrol and piceatannol biosynthesis in leaves of young seedlings, as well as increasing the expression of five *STS*-like genes in leaves of mature trees, fruit flesh, and seeds [204]. Subsequently, the same authors confirmed the increased expression of *STS*-like genes under high temperature; however, they did not find a significant increase in the accumulation of total stilbenes in stems and roots of one-year-old plants, suggesting an influence from post-transcriptional regulation on stilbene biosynthesis [205].

The impact of temperature on stilbene biosynthesis was also investigated in grapevines on whole plants, post-harvested fruits, and cultured cells. In 2017, Pastore and co-workers [206] analyzed the entire transcriptome of the berry skin in *V. vinifera* cv. Sangiovese during ripening under high temperature or low temperature regimes characterized respectively by 26 and 21 °C as average and 42 and 35 °C as maximum daily air temperature. They observed an inhibitory effect of high temperatures on stilbene biosynthesis, in contrast to low temperatures that induced the expression of several members of *STS* and *PAL* multigene families, indicating the activation of stilbene biosynthesis. A coordinated expression of *STS* and *PAL* has been often observed in grape berries, suggesting that several enzymatic steps in the stilbene biosynthetic pathway are co-regulated [279]. An inhibitory effect of high temperature on the stilbene biosynthetic pathway in grapevine was also reported by Rienth et al. [280] and Wang et al. [207].

Several studies investigated the impact of post-harvest temperature treatment on stilbene biosynthesis. Grapes stored at 0 °C showed a cultivar-dependent modulation in the expression of *STS* genes, as well as in the accumulation of resveratrol, resveratrol-glucoside, *trans-*piceatannol, z-miyabenol, and pallidol, especially in the Red Globe cultivar [208,209]. Grape berries cv. Shiraz exposed to high temperature (40 °C) showed an increase in viniferin content, which was accompanied by a decrease in the resveratrol and piceid content [210]. It was also reported that cold storage in combination with UV-C enhanced *cis-* and *trans-*piceid content in cv. Red Globe berries stored at 4 °C, while no increase of piceids was obtained by UV-C postharvest treatment alone [196]. 

Different wilting conditions during winemaking were also investigated by Versari and co-workers [185], revealing an increase of resveratrol and *STS* mRNA under traditional (ambient temperature for 100 days), low temperature (28 °C for 15 days), and mixed temperature (45 °C for 36 h, ambient temperature for 94 d) wilting, especially with the latter method. No increase in resveratrol was reported with high temperature (45 °C for 110 h) wilting. Temperature-dependent resveratrol accumulation was observed by Houillé and colleagues [217], who reported an optimal range for *trans-*resveratrol biosynthesis at 15–20 °C, a delayed accumulation of this stilbene at 5 °C, and inhibition at -20 °C and under heat shock (65 °C for 2 h immediately after cane harvest, followed by storage at 20 °C).

Taken together, the data available to date show that temperature has a significant impact on the biosynthesis of stilbenes, which is, however, highly variable in relation to the cultivar, the biological system investigated, and the interaction with other environmental factors.

### 3.4. Wounding

It is well known that the biosynthesis of stilbenes in plants can be triggered by physical stimuli such as wounding stress. This abiotic stress has been shown to affect stilbene accumulation in *Vitaceae* [224], *Fabacee* [203], and *Pinaceae* [221]. 

Freshly pruned canes of *V. vinifera* cv. Pinot Noir showed a transient expression of *PAL* and *STS* genes, followed by a rapid increase in *trans*-resveratrol and *trans*-piceatannol, when cut in short segments (from 0.2 to 10 cm), with the highest increase in 0.5 cm-length sections [224]. According to previous studies [217], only *trans*-resveratrol and *trans*-piceatannol biosynthesis was elicited in pruned grape canes, while no increase in the content of *trans*-ε-viniferin, ampelopsin A, *trans*-miyabenol C, *cis*- and *trans*-vitisin B, hopeaphenol, and isohopeaphenol was recorded. The sequential induction of lipoxygenase (*VvLOX*, involved in jasmonic acid biosynthesis) and *VvSTS* genes suggested that the activation of stilbenoid metabolism in response to wounding stress involves the jasmonate signaling pathway [224]. 

The induction of grapevine stilbene biosynthetic genes after wounding has been reported in several studies. The genome-wide analysis carried out by Vannozzi and colleagues [60] on *V. vinifera* cv. Pinot Noir showed an increase in the transcription level of several *VvSTS* gene family members. Induction of *VvSTS* in canes from grapevine Cabernet Franc has been recorded during the first 4 weeks of storage by Houillé and co-workers [217], indicating that grapevine wood is still transcriptionally active after pruning. In 2017, Yin et al. [220] observed a significant induction by wounding stress of *VqSTS36* promoter activity in *Vitis quadrangularis* L. In 2018, Vannozzi and co-workers [218] reported that in response to wounding the transcript level of *VviSTS29*, -*41* and -*48* gradually increases, coupled with the induction of WRKY and R2R3-MYB transcription factors. Four *WRKY* genes, namely *VviWRKY03*, *VviWRKY24*, *VviWRKY43,* and *VviWRKY53*, were thus reported for being involved in the regulation of the stilbene biosynthetic pathway.

Several studies on Scots pine have shown remarkable increases in pinosylvin and pinosylvin monomethyl ether accumulation following wounding stress, associated with overexpression of *PsSTS*, as well as of genes coding for *O*-methyltransferases involved in stilbene methylation (*PsMT1* and *PsMT2*) [134,221,223].

Increases in *trans*-resveratrol accumulation coupled to overexpression of stilbene genes in response to wounding stress were also observed in *A. hypogaea* [202,203,225].

### 3.5. Biotic Stress

Stilbenes are well-known to act as chemical defense compounds against pathogen attack in plants [3]. Certain stilbene-producing plants constitutively biosynthesize high levels of stilbenes, independently from pathogen infection. However, in many species, the expression of stilbene biosynthetic genes and the production of stilbenes increase rapidly and conspicuously in response to pathogenic attack. For example, in the roots of *P. cuspidatum*, large amounts of resveratrol and stilbene glucosides are constitutively accumulated [281,282]. In Scots pine, high levels of pinosylvin and pinosylvin 3-*O*-methyl ether constitutively accumulate in the heartwood where they protect the wood against decaying fungi [283], but different stress factors including herbivore and pathogen attack can also elicit the biosynthesis of both stilbenes in sapwood and needles, where they consequently act as phytoalexins [284,285].

In grapevine, both *STS* gene expression and de novo synthesis of stilbenes are induced upon infection with different phytopathogenic fungi like *Botrytis cinerea* (gray mold) [286,287,288,289], *Plasmopara viticola* (downy mildew) [60,92,290], *Erysiphe necator* (powdery mildew) [90,291,292], *Rhizopus stolonifer* (black bread mold) [183], *Aspergillus* spp. [293,294], and *Phaeomoniella chlamydospora* (associated with Esca and Petri diseases) [295].

To date, the impact of bacterial infection on stilbene biosynthesis in grapevine has been poorly investigated. A bacterial strain belonging to the genus *Bacillus* has been found to elicit *trans*-resveratrol biosynthesis in *V. vinifera* cv. Chardonnay and *V. rupestris* in vitro-grown plantlets [296]. In 2011, Verhagen and colleagues [297] reported that different bacterial strains originating from the vineyard such as *Pantoea agglomerans* (Pa-AF2), *Bacillus subtilis* (Bs-271), *Acinetobacter lwoffii* (Al-113), and *Pseudomonas fluorescens* (Pf-CT2) can elicit *trans*-resveratrol and *trans*-ε-viniferin biosynthesis in cells and leaves of *V. vinifera* cv. Chardonnay. In 2015, Gruau and co-workers [298] investigated the ability of grapevine cv. Chardonnay plants to express immune responses at both above- and below-ground after interacting with the beneficial bacterium *P. fluorescens* (PTA-CT2). Bacterial colonization occurred exclusively in the roots and altered the plant phenotype that exhibited multiple defense responses both locally and systemically. The interaction with bacteria-induced opposite changes in stilbene levels in leaves and roots. Significant increases in the content of *trans*-resveratrol, *trans*-piceid, and *trans*-ε-viniferin were observed in the leaves, while the content of all three stilbenes significantly decreased in the roots. This suggests that this interaction plays a role in the transfer of stilbene phytoalexins to the shoot, contributing to the systemic immune response [298]. Both Verhagen et al. [297] and Gruau et al. [298] showed that defense responses triggered by the interaction with beneficial bacteria can greatly improve grapevine resistance against the fungal pathogen *B. cinerea*.

Constitutive accumulation of resveratrol has been detected in several tissues of peanut plants, albeit at extremely low concentrations. A dramatic increase in both the quantity and diversity of stilbene phytoalexins can occur in response to fungal infection in peanuts [299]. In 2008, Sobolev [300] observed that the inoculation of peanut kernels with different fungal strains belonging to the genus *Aspergillus* strongly elicits the biosynthesis of *trans*-resveratrol, *trans*-arachidin-1, *trans*-arachidin-2, *trans*-arachidin-3, *trans*-3-isopentadienyl-4,3′,5′-trihydroxystilbene, and SB-1. All tested fungal strains of *Aspergillus* species infecting peanuts activated stilbenoid production in peanut kernel, with interesting variations among different kernel layers. After 24 h of incubation, the tissues closer to the infection site accumulated all the analyzed compounds, while tissues distant from the infected area almost exclusively contained *trans*-resveratrol. After 48 h of incubation, the six stilbenes were also accumulated in areas far from the infection site, suggesting that *trans*-resveratrol serves as the building block for other stilbenoids [300].

In peanuts, as well as in grapevine, data regarding the impact of bacterial infection on stilbene biosynthesis are still scarce. To compare the inductive effects of fungi and bacteria on stilbenoid biosynthesis, two phytopathogenic fungal strains (*Botryodiplodia theobromae* LBBT HC6-1 and *B. cinerea* FCBC TN1) and two Gram-negative phytopathogenic bacterial strains (*Xanthomonas campestris* pv. *citri* XW24 and *Pseudochrobactrum asaccharolyticum*) were used to treat peanut calluses [241]. The elicitation treatments were performed with either viable or non-viable (autoclaved) microorganisms. The results showed that fungal elicitation is much more effective in inducing biosynthesis of *trans*-resveratrol and *trans*-piceatannol than bacterial elicitation, regardless of species and viability [241]. 

### 3.6. Elicitation

Plant cell and organ cultures obtained from stilbene-producing plants represent a reliable model system, both for basic research on plant defense mechanisms and for the biotechnological stilbene production, that can be induced and/or enhanced by a wide range of elicitors. The term elicitor, which was originally referred to as molecules capable of inducing the biosynthesis of phytoalexins in plants, is currently used to designate any physical and chemical factors that can trigger any kind of defense response in plants [301]. Elicitors can be classified, according to their nature, as “abiotic elicitors” or “biotic elicitors” and, according to their origin, as “exogenous elicitors” or “endogenous elicitors” [301,302]. Elicitation has mainly been investigated as a tool to enhance the in vitro production of stilbenes [40,219,233,303,304]. However, in recent years increasing attention has been paid to elicitation in both pre- and post-harvest in vivo models, as a strategy to induce the natural plant defenses against pathogens and to improve the health properties of plant foods [188,189,239,305,306,307].

Methyl jasmonate (MeJa) has been shown to be the most effective elicitor for promoting stilbene production in grapevine cell cultures [219,227,233,234,303,308,309]. However, stilbenes are mainly accumulated intracellularly and only a small fraction of them is released into the culture medium by MeJa-treated grapevine cells. Complexation with cyclodextrins (CDs) promotes the release of stilbenes into the medium [304,310]. CDs are cyclic oligosaccharides that have been reported to activate the expression of stilbene biosynthetic genes through the induction of several transcription factors in grapevine [311]. Dimethyl-β-CDs have been shown to act as elicitors capable of stimulating the biosynthesis of stilbenes, as well as promoting their release in the medium and increasing their stability [304,308]. The efficacy of the combined use of MeJa and dimethyl-β-CDs in enhancing the biosynthesis and the extracellular secretion of stilbenes in grapevine cell cultures has been demonstrated by extensive literature [228,304,311,312,313,314]. It has been recently reported that the co-treatment with MeJa and stevioside (a diterpene glycoside extracted from leaves of *Stevia rebaudiana* Bertoni), elicits the production and extracellular secretion of resveratrol and viniferins in cell cultures of *V. labruscana* cv. Campbell Early [304].

CDs have been widely exploited to enhance the production of stilbene compounds in hairy root cultures of *A. hypogaea*. Co-treatment of peanut hairy roots with CDs and MeJa induced the production and secretion in the culture medium of *trans*-resveratrol, piceatannol, *trans*-arachidin-1, and *trans*-arachidin-3 [242]. In 2019, Somboon and colleagues [315] reported that the elicitation of *A. hypogaea* hairy roots with the herbicide paraquat (1,1′-dimethyl-4,4′-bipyridinium dichloride), followed by the combination of MeJa and CDs, resulted in increased amounts of stilbene compounds like *trans-*resveratrol, *trans-*arachidin-1, and *trans-*arachidin-3. Physical elicitors have also been widely exploited for the induction of stilbenes production in peanut, as discussed by Hasan et al. [299] and Wongshaya et al. [316].

The elicitation of white mulberry (*Morus alba*) callus cultures with 2-hydroxypropyl-β-CD has been shown to improve resveratrol and oxyresveratrol production [317]. It has also been shown that the treatment of cell suspension cultures of *M. alba* with MeJa and yeast extract (YE) increases the production of oxyresveratrol and resveratrol [318]. A recent investigation has demonstrated that root cultures of *M. alba* co-treated with MeJa and YE produce relatively high levels of resveratrol, oxyresveratrol, and mulberroside A [319].

In peanut, different chemical elicitors proved to be effective in inducing the biosynthesis of stilbene phytoalexins. Induction of resveratrol by treating peanut cell suspension cultures with YE was reported by Lanz and co-workers [320]. A single elicitation treatment with sodium acetate of peanut hairy root cultures resulted in a 60-fold induction and secretion of *trans*-resveratrol into the medium after 24 h [321].

Several studies have been conducted with various elicitors to enhance stilbene production in both pre- and post-harvest grape berries. In 2015, Guerrero and colleagues [322] investigated for the first time the effect of pre-harvest UV elicitation in grapevines. Twenty-four hours after the UV-C pre-harvest treatment, they observed a 22 to 46-folds increase in *trans*-resveratrol content in table grapes cv. Red Globe. They subsequently reported that pre-harvest UV-C treatment repeated for 3 consecutive days resulted in an 86-fold increase in stilbenoid content (*trans*-resveratrol, *trans*-piceatannol, isorhapontigenin, *cis*- and *trans*-piceid, ε- and ω-viniferin) in table grapes cv. Crimson seedless [323]. 

As previously mentioned (Section 3.1), a vast literature describes the impact of UV elicitation on stilbene accumulation in post-harvest grapes [94,324]. In 2019, Segade and co-workers [325] recently investigated the impact of post-harvest ozone elicitation on the biosynthesis of stilbenes in Moscato bianco winegrapes. They found that short-term treatment with high ozone doses (60 μL/L; 48 h exposure) not only prevented the loss of stilbenes during the dehydration process but also induced the accumulation of *trans*-resveratrol and *trans*-piceatannol. Surprisingly, long-term and continuous ozone treatment did not induce *trans*-resveratrol production, but it did not negatively affect the stilbene content in winegrapes. Consequently, the use of ozone could have important applications in winemaking to produce wines with high added value considering both the possible reduction of SO_2_ addition and, under certain conditions, increased contents of stilbenes [325]. 

Ultrasonication is mechanical stress that can affect stilbene biosynthesis, dramatically increasing resveratrol levels in peanut kernels, as well as in grape skin and leaves. Recent reviews [20,326] thoroughly covered this topic, reporting an increase in *trans*-resveratrol, *trans*-piceid in peanuts [201,327], and an increase of resveratrol coupled with the up-regulation of the resveratrol synthase (RS) gene in grape skins and leaves [328] treated with ultrasound. It was also reported that short-term exposure to low power ultrasonication is more effective in eliciting resveratrol accumulation. More recently, Yu and co-workers [329] obtained similar results in peanut sprouts, which responded to ultrasound treatment with an increase in *trans*-resveratrol, accompanied by a slight increase in total sugars and a remarkable decrease in crude fat and peanut allergenic proteins. Despite these promising results, the use of ultrasonication as a physical elicitor to increase the biotechnological stilbene production in vitro has been poorly investigated. The first published study on this topic dates to 2012 when Santamaria and collaborators [219] reported the accumulation of *trans*-resveratrol, *trans*-piceid, *trans*-ε-viniferin, *trans*-δ-viniferin, and *cis*-viniferin in grapevine cv. Alphonse Lavallée cell suspensions in response to low-energy ultrasounds.

### 3.7. Other Environmental Factors

Stilbene biosynthesis is affected by a wide array of abiotic environmental factors besides the ones mentioned above, and extensive literature has been produced regarding this subject. Past reviews identified, aside from UV, light, wounding and temperatures, drought, ultrasonication, ozone, salinity, pesticides, and soil nutrient content as important abiotic stress sources capable of modulating stilbene biosynthesis [3,5,6,20,330].

Water stress has been shown to produce conflicting results on stilbene biosynthesis in grapevine, with either an increase [96,331] or decrease [332,333] in stilbene content and different behaviors among resveratrol, piceid, and viniferin content in different cultivars, suggesting a pivotal role for genotype [334]. The diversity in polyphenol composition and its changes in response to drought has been recently investigated on a large panel of grapevine cultivars by Pinasseau et al. [335].

The effects of ozone on stilbene biosynthesis, on the other hand, have been known for several decades. Ozone was observed to increase STS activity, as well as the biosynthesis of pinosylvin and pinosylvin 3-methyl ether in Scots pine seedlings [336]. It was also reported to induce an augmented transcript level of the *PMT* gene in Scots pine needles [221]. Sitka spruce (*Picea sitchensis* (Bong.) Carr.) stem bark tissues have also been observed reacting to ozone treatment with an increase in resistance to pathogens, although no alterations in the levels of the stilbene glucosides astringin and isorhapontin were recorded [337].

*Vitis* spp. were also investigated through cell cultures [338] and post-harvest treatment [183,191], recording general increases in *trans*-resveratrol, piceatannol, pterostilbene, viniferins, and increases in *STS* expression in response to ozone (although only on ozone resistant phenotypes). Further investigations of *STS* expression in transgenic tobacco plants in combination with its promoter (*Vst1*) and the β-glucuronidase (GUS) reporter gene resulted in 11-fold GUS expression following a single ozone pulse and induction of the Vst1 promoter in several tissues [339]. Recent reviews also covered the elicitation of stilbenes due to ozone [5,20], a topic that was later expanded by Ghimire and colleagues [340] observations published in 2019 regarding the combined effects of elevated ozone, temperature, and nitrogen on stilbene concentrations in Scots pine. They reported that warming does suppress the induction of some stilbene compounds/derivatives in ambient ozone levels.

Another important abiotic stress is salinity. Salt stress represents a devastating constraint to plants’ development processes and physiological homeostasis, causing membrane disorganization, cytoplasm alkalinization, ROS induction, transport perturbations, tissue proliferation, and perturbations to photosynthesis, ionic and ionic-related channels [95].

Sodium chloride (NaCl) salt stress is known to strongly delay the rapid induction of *STS* and resveratrol production in *V. rupestris* (Scheele) cell cultures, with a late increase in stilbenes. *V. riparia* (Michx.), instead, does not seem to respond to salt stress [341].

A recent experiment on *V. vinifera* plantlets subjected to NaCl treatment revealed that the salt-tolerant cv. Razegui did not show important variations in stilbenes biosynthesis, while the salt-sensitive cv. Syrah showed an increase in *trans*-resveratrol, *trans*-piceid, and *cis*-piceid, probably to cope with the higher oxidative disturbance [95]. Resveratrol seems to possess an important role in salt adaptation, as it was found that the application of resveratrol in combination with α-tocopherol to *Citrus aurantium* L. seedlings reduces NaCl membrane permeability, lipid peroxidation, and pigments degradation, besides reducing H_2_O_2_ accumulation in leaves and restoring the reduction of photosynthesis induced by NaCl [342].

Different salts also affect resveratrol production in grapevine. In 2013, Cai and co-workers [343] analyzed the effects of cobalt chloride (CoCL_2_), silver nitrate (AgNO_3_), and cadmium chloride (CdCl_2_) on *V. vinifera* cv. Gamay Fréaux cell cultures. They reported that cobalt ions at 5.0, 25, and 50 μM concentrations and silver and cadmium ions at 5.0 μM concentration stimulated a 1.6-fold increase in 3-*O*-glucosyl-resveratrol without suppressing cell growth or compromising cell viability. In contrast, higher concentrations of silver and cadmium remarkably reduced cell viability.

To further expand this topic, we remand to a recent excellent review from Hasan and Bae [20].

## 4. Conclusions and Future Prospects 

The study of stilbenes is of crucial importance for basic research, to understand the biological role of these metabolites in plant defense from biotic and abiotic stress. The interest towards stilbenes also comes from applied research, mainly due to the numerous bioactivities of these compounds and the consequent potentials in the nutraceutical, cosmeceutical, and pharmaceutical fields. The present review arises in this context, dealing with the most recent knowledge of the stilbene biosynthetic pathway and the environmental factors that affect their biosynthesis and accumulation in different plant species, both *in planta* and in in vitro systems. Given the vastness of the topic and the abundance of scientific literature available to date, this review is not intended to be comprehensive. Some issues concerning the study of stilbenes, albeit of great interest, such as their biological function, pharmacological activity, and distribution in nature and in plant foods have not been explored, because they have been the subject of recent excellent reviews [2,3,4,22,170]. Another topic of great interest, extensively covered by a recent review by Dubrovina and Kiselev [5], consists in the study of the molecular mechanisms by which environmental stimuli regulate the stilbene biosynthetic genes. In recent years, increasing efforts have been devoted by different research groups to expand knowledge on this topic, which is leading to the emergence of interesting new knowledge. Therefore, we expect to have in the near future a more detailed model of the molecular mechanisms that link the perception of environmental stimuli to the expression of genes responsible for the biosynthesis of stilbenes. Furthermore, we expect research efforts to shed light on the link between the biosynthesis of stilbenes and environmental factors that have so far been poorly studied, such as viruses, bacteria, herbivores, UV-A, and ionizing radiation.

## Figures and Tables

**Table 1:** Induction of stilbene biosynthetic gene expression and stilbene accumulation by environmental factors.

Species/Cultivar/Variety	Treatment/s	Metabolites	Results	Reference
*Vitis vinifera* cvs. Alphonse Lavallée, Dan Ben-Hanna, Dabuki, Early Superior, Flame seedless, Kishmish, Muscat Hamburg, Perlette, Spring Blush, Superior, Thompson seedless, Zeiny, Gamay, Gamaret, Pinot, Shasla	Irradiation of grape berries with UV-C or inoculation of grape berries with *Rhizopus stolonifer*	Stilbenes (resveratrol and pterostilbene)	Increased stilbene accumulation, greater with UV-C compared to fungal inoculum	[183]
*V. vinifera* cv. Napoleon	Irradiation of grape berries with UV-C or UV-B	Stilbenes (resveratrol and piceid); anthocyanins; flavonoids; hydroxycinnamic acids (caffeoyltartaric acid and chlorogenic acid)	Increased stilbene accumulation, greater with UV-C compared to UV-B (3 and 2-fold, respectively)	[184]
*V. vinifera* cv. Corvina	Irradiation of grape berries with UV-B and wilting at different temperatures	Stilbenes (*trans* and *cis*-resveratrol, *trans* and *cis*-piceid); total polyphenols, flavonoids, anthocyanins, catechins, and proanthocyanidins	Enhanced stilbene accumulation and *STS* gene expression	[185]
*V. vinifera* cvs. Black Corinth and Flame seedless	Irradiation of grape berries with UV-C	Resveratrol; total anthocyanins	Greater resveratrol increase (4-fold) in cv. Flame Seedless. Lower increase in cv. Black Corinth. Negative relationship between resveratrol synthesis and anthocyanin concentration	[186]
*V. vinifera* cv. Flame seedless, Red Globe, Crimson seedless, Napoleon, Superior seedless, Moscatel Italica, Dominga	Irradiation of grape berries with UV-C	*Trans*- and *cis*-resveratrol, *trans*-piceatannol, *trans*-piceid, *trans*-astringin, α-viniferin, ε-viniferin	Increased stilbene concentration, with higher accumulation of *trans-*resveratrol, *trans-*piceatannol, and viniferins	[187]
*V. vinifera* cv. Monastrell	Irradiation of grape berries with UV-C, followed by traditional maceration	Stilbenes (*trans*-resveratrol, *trans*-piceatannol); anthocyanins; flavonols; flavanols (total catechins); hydroxycinnamic acids (*p*-coumaroyltartaric acid)	Increased in *trans*-piceatannol and *trans*-resveratrol content (1.5 and 2-fold, respectively) in wines without impacting standard oenological parameters	[188]
*V. vinifera* cvs. Tempranillo, Cabernet-Sauvignon, Merlot, Syrah, Monastrell, Garnacha, Cariñena	Irradiation of grape berries with UV-C	*Trans-*resveratrol, *trans-*piceatannol, α-viniferin, ε-viniferin	Increased concentrations of *trans-*resveratrol, *trans-*piceatannol, and viniferins in grape skins of all varieties, except Monastrell, in which only *trans-*piceatannol concentration increased	[189]
*V. vinifera* cv. Superior	Irradiation of grape berries with UV-C	*Trans*-resveratrol, *trans-*piceid, *trans-*piceatannol, viniferins (resveratrol dehydrodimers and dehydrotrimers)	Increased *trans*-resveratrol accumulation (10-fold); induction of *trans-*piceid, *trans-*piceatannol, and viniferins (not detected in control grapes)	[190]
*V. vinifera* cv. Superior	Comparison of UV-C and ozone (O_3_) treatments on grape berries	*Trans*-resveratrol, piceatannol, and viniferins (resveratrol dehydrodimers and dehydrotrimers)	Increased accumulation of stilbenes after both UV-C and O_3_ treatments. O_3_ more effective than UV-C in inducing the accumulation of viniferins	[191]
*V. vinifera* cv. Superior	Irradiation of grape berries with UV-C, followed by maceration with Na_2_S_2_O_5_ and enzymes	Stilbenes (*trans*-resveratrol; *trans*-piceid; *trans*-piceatannol, viniferins); hydroxycinnamic acids; flavonols; flavanols (catechins and procyanidins)	Increased stilbene concentration (35-fold) in grape juice under optimum conditions (maceration for 2 h at 45 °C with 0.2% Na_2_S_2_O_5_ using UV-C-treated grape berries)	[192]
*V. vinifera* cv. Red Globe	Irradiation of grape berries with UV-B nanosecond laser pulses	*Trans-*resveratrol	Increased *trans-*resveratrol accumulation (6-fold) in grape berries subjected to a resonant wavelength of the compound (302.1 nm)	[193]
*V. vinifera sylvestris* var. V9. V15, V16; *V. vinifera sativa* var. Merlot, Syrah, Graciano, Tempranillo, Palomino fino, Palomino negro, Tintilla de Rota; *V. vinifera sativa* hybrid Orion, Regent	Irradiation of grape berries with UV-C	*Trans-*resveratrol, piceatannol, ε-viniferin, δ-viniferin	Increased stilbene concentration, with differences depending on variety and campaign, but not on subspecies	[194]
*V. vinifera × V. labrusca* cv. Kyoho	Irradiation of grape berries with UV-C and storage at different temperatures (0 °C or 20 °C)	Resveratrol	Increased resveratrol concentration, especially in UV-treated grapes stored at high temperature	[195]
*V. vinifera* cv. Redglobe	Irradiation of grape berries with UV-C and storage at different temperatures (25 °C or 4 °C)	Stilbenes (*trans-*resveratrol, *cis*- and *trans*-piceid); flavonols; anthocyanins; flavanols (catechins)	Increased concentration of *cis*- and *trans*-piceid after UV-C treatment and cold storage	[196]
*V. vinifera* cv. Crimson	Treatment of grape berries with UV-C and chitosan, followed by storage at different temperatures	*Trans-*resveratrol	Increased resveratrol content in grapes and lower susceptibility to fungal decay after UV-C treatment combined with chitosan coating followed by storage at 20 °C for 24 h before refrigerated storage	[197]
*V. amurensis* cv. Tonghua-3	Treatment of grape berries with UV-C	*Trans-* and *cis-*resveratrol	Increased accumulation of stilbene compounds, up-regulation of multiple *STS* genes, down-regulation of *CHS* genes	[198]
*V. vinifera* × *V. labrusca* cv. Summer Black	Treatment of grape berries with UV-B or UV-C	Stilbenes (*trans-*resveratrol, *trans-*piceid); gallic acid; hydroxycinnamic acids (caffeic acid, *trans*-ferulic acid); flavanols [(+)-catechin, (−)-epicatechin, epicatechin gallate]	Increased accumulation of phenolic compounds and *STS* gene expression, more induced by UV-C than UV-B	[199]
*V. vinifera* cv. Kyoho	Irradiation of grape berries with UV-B	*Trans-*resveratrol, *trans-*scirpusin A, *trans-*ε-viniferin, *trans-*δ-viniferin, *trans-*pterostilbene	Increased production of the analyzed stilbenes, up-regulation of stilbene biosynthetic genes	[200]
*Arachis hypogaea* cv. Georgia green	Treatment of peanuts with UV-C or ultrasonication	*Trans-*resveratrol, *trans-*piceid	Increased resveratrol, piceid, and total stilbene concentration, more induced by ultrasound than UV-C	[201]
*A. hypogaea* var. Jinpoong	Leaves subjected to UV-C, wounding, paraquat, H_2_O_2_, salicylic acid, jasmonic acid ethephon, abscisic acid	Resveratrol	Maximum resveratrol increases in response to UV (over 200-fold), followed by paraquat (20-fold) and wounding, H_2_O_2_, salicylic acid, jasmonic acid, and ethephon (between 2- and 9-fold)	[202]
*A. hypogaea* Georgia green	Treatment of peanuts with UV-C	*Trans-*resveratrol	Increased *trans-*resveratrol accumulation (10-fold)	[203]
*Gnetum parvifolium*	Treatment of 1-year-old seedlings with high temperature (40 °C) and UV-C treatments	Resveratrol and piceatannol	Both high temperature and UV-C strongly induce the expression of *PAL*, *C4H-*, *4CL-*, and *STS-like* genes, but only UV-C enhance stilbene accumulation	[204]
*Gnetum parvifolium*	Treatment of 1-year-old seedlings with high temperature (40 °C) and UV-C treatments	Resveratrol and piceatannol	Both high temperature and UV-C strongly induce the expression of *PAL*, *C4H, 4CL*, *STS*, and *CYP* genes. High temperatures do not affect stilbene accumulation in stems but decrease stilbene concentration in roots at 3 h. UV-C irradiation induces total stilbene accumulation in stems but not in roots.	[205]
*Pinus sylvestris*	Treatment of needles from 5-years-old plantlets with UV-C	Pinosylvin and pinosylvin monomethylether	Induction of *PMT2* expression	[134]
*V. vinifera* cv. Sangiovese	Potted vines grown in air-conditioned greenhouses under high temperature or low temperature regimes (26 and 21 °C as average and 42 and 35 °C as maximum air daily temperature, respectively)	Stilbenes	Increased expression of *STS* and *PAL* genes under low temperatures	[206]
*V. vinifera* cv. Cabernet Sauvignon	Treatment of cell suspension cultures with high temperature (38 °C) or low temperature (16 °C) and CuSO_4_	Stilbenes	Downregulation of *STS* expression under both low and high temperature and upregulation of *STS* expression in response to CuSO_4_	[207]
*V. vinifera* cv. Cardinal	Treatment of grape berries with low temperature (0 °C) and high CO_2_ levels (20%)	*Trans*-resveratrol; total anthocyanins	Low temperature reduces *trans*-resveratrol content in both treated and non-treated grapes, although the decrease is higher in CO_2_-treated grapes	[208]
*V. vinifera* cvs. Dominga, Superior seedless, Autumn Royal, Red Globe	Treatment of grape berries with low temperature (0 °C) and high CO_2_ levels (3 days)	Resveratrol, resveratrol-glucoside, *trans*-piceatannol, z-miyabenol, pallidol	Stilbene accumulation in response to low temperature and CO_2_ is cultivar dependent. High CO_2_ levels activate stilbene pathways in cv. Dominga. Low temperature increase stilbenes biosynthesis in cv. Red Globe. Stilbene accumulation is independent of the atmosphere storage in cvs. Superior Seedless and Autumn Royal	[209]
*V. vinifera* cv. Shiraz	Treatment of grape berries with high light (2500 μmol m^−2^ s^−1^), high temperature (40 °C), oxidative stress (120 μM menadione), 3.026 mM abscisic acid, and 200 μM jasmonic acid (JA)	Resveratrol, piceid, and viniferin	At the pre-veraison stage, an increase in anthocyanins levels is accompanied by a declining stilbene accumulation in response to JA, menadione, and high light. At the veraison stage, mild change in anthocyanin levels in response to all the treatments is accompanied by stilbene accumulation	[210]
*V. vinifera* cv. Barbera	Treatment of cell suspensions with red LED light (1.34 μE m^−2^ s^−1^, 625 ± 10 nm) and 10 μM methyl-jasmonate (MeJa)	Stilbenes (*cis*- and *trans-*piceid, *cis*- and *trans-*resveratrol, *cis*- and *trans-*resveratroloside); catechins; anthocyanins	Strong increase in total stilbenes induced by MeJa, whose effect is enhanced by a red light. Increase in total anthocyanins in response to MeJa, used alone or in combination with a red light. Decrease in catechins under red light; increase in response to MeJa alone or in combination with red light	[211]
*V. labruscana* cvs. Campbell Early and Kyoho	Treatment of grape berries and leaves with fluorescent white light and purple, blue, and red LED lights	*Cis*- and *trans-*resveratrol, *cis*- and *trans-*piceid, *cis*- and *trans-*piceatannol	Increased accumulation of stilbenes (mainly *trans*- and *cis*-piceid) and induction of stilbene biosynthetic genes in response to red and blue LED light	[212,213]
*V. vinifera* cv. Negramaro	Light-exposed and dark-maintained cell cultures	*Trans-*resveratrol, *trans-*piceid, *cis*-ε-viniferin, *trans*-ε-viniferin, *trans*-δ-viniferin	Higher levels of *trans*-resveratrol and viniferins under darkness; higher levels of *trans*-piceid under light	[214]
*V. vinifera* cv. Shahani	High-level white light irradiation (10,000 lux) and MeJa (25, 50, 100 and 200 μM)	Stilbenes (*trans-*resveratrol, *trans-*piceid); total phenols; total flavonoids	Inhibitory effect of high light on stilbene biosynthesis; 50 μM MeJa is optimal for efficient production of total phenols, flavonoids, and stilbenes	[215]
*V. vinifera* cv. Malvasia; *V. rupestris* Du Lut	Light-exposed and dark-maintained cell cultures	*Trans-*resveratrol, *trans-*piceid, *trans*-ε-viniferin, *trans*-δ-viniferin	Increase in stilbene content under light conditions	[40]
*Arachis hypogaea*	White LED light and UV-C radiation during peanut germination	Stilbenes (resveratrol, piceid, piceatannol); total phenols; total flavonoids	White light significantly induces stilbene accumulation by upregulating the expression of genes and enzymes involved in the stilbene biosynthetic pathway. UV-C is more effective than white light in promoting stilbene accumulation	[216]
*V. vinifera* cvs. Cabernet Franc, Chardonnay, Chenin, Malbec (Côt), Gamay, Grolleau, Pinot Noir, Sauvignon Blanc	Wounding (stem pruning)	*Trans-*resveratrol, *trans*-piceatannol, *trans*-ε-viniferin, ampelopsin A, *trans*-miyabenol C, *cis*- and *trans*-vitisin B, hopeaphenol, isohopeaphenol	Induction of *trans*-resveratrol and *trans*-piceatannol during the first 6 weeks of storage at 20 °C	[217]
*V. vinifera* cv. Pinot Noir	Wounding (leaf discs)	Stilbenes	Increase in transcription levels of several *STS* genes	[60]
*V. vinifera* cv. Pinot Noir	Wounding (leaf discs)	Stilbenes	Increased transcript level of *VviSTS29*, -*41,* and -*48*, coupled with the induction of WRKY and R2R3-MYB transcription factors	[218]
*V. vinifera* cv. Alphonse Lavallée	Mechanical stress (low-energy ultrasound) alone or in combination with MeJa on cell suspension cultures	*Trans-*resveratrol, *trans-*piceid, *trans*-ε-viniferin, *trans*-δ-viniferin	Increase in *trans-*ε-viniferin production in response to ultrasounds. Increase in *trans-*δ-viniferin in response to ultrasound and MeJa co-treatment	[219]
*V. quinquangularis*	Wounding, exogenous stress-associated hormones, and biotic stress in leaves of transgenic tobacco transformed with *VqSTS36* promoter fused to the *GUS* reporter gene	Stilbenes	Induction of *VqSTS36* promoter activity in response to wounding, salicylic acid, and inoculation with the phytopathogenic fungus *Erysiphe cichoracearum*	[220]
*Pinus sylvestris*	Wounding of stem-phloem alone or in combination with fungal infection	Stilbenes	Transient increase in *STS* and *PMT* expression in response to wounding, more pronounced with wounding in combination with fungal inoculation	[221]
*P. sylvestris*	Wounding of seedlings	Pinosylvin and pinosylvin monomethyl ether	Upregulation of stilbene biosynthetic genes including *PMT2* during heartwood formation and in response to stress	[222]
*P. sylvestris*	Wounding and infection of seedlings with *Heterobasidion parviporum* or *H. annosum*	Pinosylvin and pinosylvin monomethylether	Significantly higher amounts of stilbenes 10 days after treatment. Greater increase in infected than in just wounded samples	[223]
*P. sylvestris*	Wounding 5-years-old seedlings	Pinosylvin and pinosylvin monomethylether	Induction of *PMT2*	[134]
*V. vinifera* cv. Pinot Noir	Mechanical wounding on freshly pruned canes	*Trans*-resveratrol and *trans*-piceatannol	Transient expression of *PAL* and *STS* genes, followed by a rapid accumulation of stilbenes	[224]
*Arachis hypogaea*	Wounding stress (cotyledons)	Resveratrol, arachidin-3, arachidin-4	Induction of all analyzed stilbenes	[225]
*A. hypogaea*	Wounding stress (size reduction, grinding, chopping, slicing, ultrasound)	*Trans-*resveratrol	Slicing produces the highest increase of *trans*-resveratrol accumulation	[203]
*Pinus sylvestris*	Ozone fumigation (saplings grown in phytotron)	Stilbenes	Enhanced *STS* and *PMT* transcript levels in needles but not in healthy phloem	[221]
*V. quinquangularis* (accession Shang-24; powdery mildew (PM) resistant); *V. pseudoreticulata* (accession Hunan-1; PM susceptible)	Infection by *Uncinula necator* (sin. *Erysiphe necator*)	Stilbenes	*VqSTS36* transcript levels increase substantially following PM infection	[220]
*V. vinifera* cv. Barbera	Elicitation of cell suspension cultures with salicylic acid, Na-orthovanadate, jasmonates, chitosan, D-glucosamine, N-acetyl-D-glucosamine, ampicillin, rifampicin	*Trans-* and *cis*-resveratrol	Induction of *ex-novo* synthesis of stilbenes stilbene synthase protein by MeJa and chitosan	[226]
*V. vinifera* cv. Gamay Fréaux var. Teinturier	Elicitation of cell suspension cultures with MeJa in combination with sucrose	Stilbenes (*trans-*resveratrol and piceids); total anthocyanins	Induction of *PAL*, *CHS*, *STS*, UDP-glucose: flavonoid-*O*-glucosyltransferase, proteinase inhibitor and chitinase gene expression. Enhanced accumulation of piceids and anthocyanins in cells, and *trans*-resveratrol and piceids in culture medium	[227]
*V. vinifera* cv. Monastrell albino	Elicitation of cell suspension cultures with MeJa and cyclodextrin (CDs) used independently or in combination	*Trans*-resveratrol	Induction of stilbene biosynthetic gene expression by MeJa and CDs when used independently. Enhanced *trans*-resveratrol production in CDs-treated cells but not in MeJa-treated cells	[228]
*V. vinifera* cv. Barbera	Elicitation of cell suspension cultures with chitosan	*Trans*- and *cis*-resveratrol	Induction of *trans*-resveratrol production and STS gene expression	[229]
*V. vinifera* cvs. Red Globe and Michele Palieri	Elicitation of calli with MeJa	*Trans*-piceid, resveratrol glucoside, *cis*-piceid, resveratrol diglucoside, resveratrol dimer monoglucosides, resveratrol dimer diglucosides, resveratrol dimer triglucosides, resveratrol dimer tetraglucosides, picetannol monoglycosylated, picetannol diglycosylated	Enhanced production of stilbenes, mainly *trans*-piceid and ε-viniferin	[230]
*V. vinifera* cv. Isabelle	Elicitation of calli with biotic (fungal extract of *Fusarium oxysporum*) and abiotic (mannitol, abscisic acid, jasmonic acid) elicitors	*Trans*-resveratrol	Optimum accumulation of *trans*-resveratrol with a combined treatment of mannitol (2 mM) and jasmonic acid (40 µM)	[231]
*V. vinifera* cv. Barbera	Elicitation of cell suspension cultures with chitosan	Mono-glucosylated derivatives resveratrol (*trans-* and *cis*-piceid and *trans*- and *cis*-resveratroloside)	Increased in *trans*-resveratrol endogenous accumulation and decreased release into the culture medium. *De-novo* synthesis and/or accumulation of STS proteins. No influence on *cis*-resveratrol and on resveratrol mono-glucosides	[232]
*V. vinifera* cv. Italia	Elicitation of calli and cell suspension cultures with MeJa, jasmonic acid or chitosan	*Trans*-resveratrol, piceid *trans*-δ-viniferin, *trans*-ε-viniferin	Induction of *trans*-resveratrol, piceid, and viniferins by jasmonates. Jasmonic acid enhances simultaneously δ- and ε-viniferin biosynthesis, whereas MeJa stimulates preferentially δ-viniferin production.	[233]
*V. vinifera* cv. Monastrell	Elicitation of cell suspension cultures with MeJa, cyclodextrins, and UV-C used independently or in combination	*Trans*-resveratrol	Highest increase in *trans*-reveratrol production was obtained with the combined use of MeJa, cyclodextrins, and an optimal sucrose concentration. Greatest release of *trans*-resveratrol into the culture medium is achieved with the combined use of MeJa, cyclodextrin, and UV-C	[234]
*V. vinifera* cv. Gamay Fréaux	Elicitation of cell suspension cultures with indanoyl-isoleucine (In-Ile), N-linolenoyl-l-glutamine (Lin-Gln), and insect saliva (from *Manduca sexta* larvae)	3-*O*-Glucosyl-resveratrol; 4-(3,5-dihydroxy-phenyl)-phenol; total anthocyanins	Increased accumulation of phenolic acids, particularly 3-*O*-glucosyl-resveratrol, in response to In-Ile, Lin-Gln, and saliva	[235]
*V. vinifera* cv. Hongbaladuo; *V. vinifera × V. amurensis* cv. Beihong	Treatment of leaves and berries with CaCl_2_ and UV-C used alone or in combination	*Cis-* and *trans*-resveratrol	Increased resveratrol content with single treatments, greater increase with combined treatment	[236]
*V. vinifera* cv. Gamay Fréaux	Elicitation of cell suspension cultures with jasmonic acid, salicylic acid, β-glucan, and chitosan	Stilbenes (*trans*-resveratrol and *trans*-piceid); total anthocyanins	Increased resveratrol production with co-treatment with jasmonic acid and β-glucan	[237]
*V. vinifera* cv. Negramaro	Elicitation of cell cultures with chitosan, MeJa, jasmonic acid, coronatine, and 12-oxo-phytodienoic acid	*Trans-*resveratrol, *trans-*piceid, *cis*-ε-viniferin, *trans*-ε-viniferin, *trans*-δ-viniferin	MeJa is the most effective in inducing *trans-*resveratrol the biosynthesis, while 12-oxo-phytodienoic acid, jasmonic acid, and coronatine are the most effective in inducing the biosynthesis of viniferins	[214]
*V. vinifera* cv. Monastrell	Elicitation cell suspension cultures with cyclodextrins and coronatine	*Trans-*resveratrol	Induction of stilbene biosynthetic genes by cyclodextrins and/or coronatine. Maximum level of *trans*-resveratrol production and secretion into the culture medium with co-treatment with 50 mM cyclodextrins and 1 μM coronatine	[238]
*V. vinifera* cv. Tempranillo	Foliar application of MeJa, chitosan, and yeast extract	Stilbenes (*trans-* and *cis*-piceid and *trans*- and *cis*-resveratrol); flavonols; anthocyanins; hydroxybenzoic acids; hydroxycinnamic acids	MeJa and yeast extract improve both grape and wine anthocyanin content. Stilbene content is clearly improved by yeast extract	[239]
*Vitis vinifera* L. cv. Kalecik Karası	Elicitation of grape berries with ultrasound	*Trans*-resveratrol	About 20-fold increase in trans-resveratrol content in grape skin	[240]
*Arachis hypogaea* cv. Tainan No. 14	Elicitation of calli with bacteria and fungi (both viable and autoclaved) or with chitin	*Trans*-resveratrol and *trans*-piceatannol	Induction of stilbene biosynthesis by fungi (both viable and autoclaved) and chitin	[241]
*A. hypogaea* cv. Hull line 3	Elicitation of hairy root cultures with MeJa and methyl-β-cyclodextrin	Trans-resveratrol, *trans*-piceatannol, *trans*-arachidin-1 and *trans*-arachidin-3	Co-treatment with MeJa and cyclodextrin led to high levels of stilbenes in the culture medium	[242]
*Arachis hypogaea* cv. Georgia green	Treatment of peanuts with ultrasonication or UV-C	*Trans-*resveratrol, *trans-*piceid	Increased resveratrol, piceid, and total stilbene concentration, more induced by ultrasound than UV-C	[201]

## Data Availability

Not applicable.

## Abbreviations

4CL	4-Coumarate:CoA ligase
CDs	Cyclodextrins
CHS	Chalcone synthase
CNL	Cinnamate:CoA ligase
C4H	Cinnamate 4-hydroxylase
DPS	Dihydropinosylvin synthase
GFP	Green fluorescent protein
GPP	Geranyl diphosphate
GUS	β-Glucuronidase
LED	Light emitting diode
LOX	Lipoxygenase
MeJa	Methyl jasmonate
OGlu	O-β-D-glucopyranoside
OMT	O-Methyltransferase
PAL	Phenylalanine ammonia-lyase
Phe	Phenylalanine
PKSs	Polyketide synthase superfamily
PMT	Pinosylvin O-methyltransferase
PS	Pinosylvin synthase
PTAL	Bifunctional phenylalanine/tyrosine ammonia-lyase
ROMT	Resveratrol O-methyltransferase
ROS	Reactive oxygen species
RS	Resveratrol synthase
STS	Stilbene synthase
TAL	Tyrosine ammonia-lyase
Tyr	Tyrosine
UV	Ultraviolet radiation
